# Experimental duration determines the effect of arbuscular mycorrhizal fungi on plant biomass in pot experiments: A meta-analysis

**DOI:** 10.3389/fpls.2022.1024874

**Published:** 2022-11-03

**Authors:** Mingsen Qin, Lei Li, Jean‐Pascal Miranda, Yun Tang, Bo Song, Maria Kathleen Oosthuizen, Wangrong Wei

**Affiliations:** ^1^ Key Laboratory of Southwest China Wildlife Resources Conservation (Ministry of Education), China West Normal University, Nanchong, China; ^2^ Department of Biology, University of York, York, United Kingdom; ^3^ Mammal Research Institute, University of Pretoria, Pretoria, South Africa

**Keywords:** Arbuscular mycorrhizal fungi, effect size, plant biomass, experimental duration, root/shoot ratio, pot size

## Abstract

Arbuscular mycorrhizal fungi (AMF) play various important roles in promoting plant growth. Numerous environmental and evolutionary factors influence the response of plants to AMF. However, the importance of the individual factors on the effects of AMF on plant biomass is not clearly understood. In this study, a meta-analysis using 1,640 observations from 639 published articles related to the influence of AMF on the plant shoot, root, and total biomass was performed; 13 different experimental setting factors that had an impact on the influence of AMF and their importance were quantitatively synthesized. The meta-analysis showed that AMF had positive effects on the plant shoot, root, and total biomass; moreover, the experimental duration, plant root-to-shoot ratio (R/S), AMF root length colonization, plant family, pot size, soil texture, and the soil pH all influenced the effects of AMF on the shoot, root, and total biomass. In addition, the plant root system and plant functional type had impacts on the effect of AMF on shoot biomass; AMF guild also impacted the effect of AMF on root biomass. Of these factors, the experimental duration, plant R/S, and pot size were the three most important predicting the effects of AMF on the plant shoot, root, and total biomass. This study comprehensively assessed the importance of the different factors that influenced the response of plants to AMF, highlighting that the experimental duration, plant R/S, and pot size should be taken into consideration in pot experiments in studies of the functions of AMF. Multiple unfavorable factors that may obscure or confound the observed functions of AMF should be excluded.

## Introduction

Arbuscular mycorrhizal fungi (AMF) are soilborne fungi that associate symbiotically with the roots of terrestrial plants; 71% of vascular plant roots harbor AMF (Brundrett and Tedersoo, 2018). Typically, AMF improve the plant nitrogen and phosphorus contents (Hodge et al., 2010; Selosse and Rousset, 2011), promote plant growth (Wagg et al., 2011), and enhance the plant’s resistance to pathogens (Qin et al., 2021) and tolerance to stress (Begum et al., 2019). Numerous studies have demonstrated the influences of plant type, plant nutrient stoichiometry (especially the nitrogen/phosphorus ratio, N/P), and environmental factors (particularly soil fertility) on the effect of AMF on plant growth (Hoeksema et al., 2010; Johnson, 2010; Delavaux et al., 2017; Zhang et al., 2019). It is essential to quantitatively determine the importance of each of these biotic and abiotic factors on the effect of AMF on plant growth for a more comprehensive understanding of the functions of AMF and for their more efficient application in ecosystems.

The percentage of AMF root length colonization (RLC) is the primary indicator of the abundance of AMF within plants or ecosystems and directly determines the degree of the symbiotic relationship and nutrient exchange ratio between plants and AMF (Batastini et al., 2021). Numerous studies on the environmental changes and temporal variations that influence AMF emphatically analyzed their impact on RLC (Johnson et al., 2010; Liu et al., 2012; Jiang et al., 2018; Vieira Junior et al., 2020). However, many quantitative studies have overlooked the association between RLC and the functions of AMF (Hoeksema et al., 2010; Lehmann et al., 2014; Leifheit et al., 2014; Lehmann and Rillig, 2015; Zhang et al., 2019). Although Treseder (2013) investigated the relationship between RLC and the effects of AMF on plant growth using a meta-analysis, this study lacked a comprehensive comparison with other factors, such as the host type and soil texture. Therefore, it is pertinent to investigate the importance of RLC in conjunction with multiple factors affecting the functions of AMF.

Another gap in our knowledge is the relationship of the functions of AMF with the plant biomass allocation strategy (as represented by the root-to-shoot ratio, R/S). AMF are known to help plants absorb numerous primary nutrients and non-nutrients from the soil (Hodge et al., 2010; Bücking et al., 2012; Delavaux et al., 2017). According to the optimal partitioning theory, a plant allocates more biomass to the organs for the most limiting resources (Johnson and Thornley, 1987), for example, to the shoot for acquiring light or CO_2_ and to the root for acquiring water or nutrients. A high plant R/S indicates that the plant allocates an appreciable proportion of its biomass to the roots to promote the acquisition of soil resources; therefore, plants with a high R/S may have greater dependence on AMF compared to plants with a low R/S in terms of absorbing soil resources (Kiers et al., 2011; Selosse and Rousset, 2011). A low plant R/S indicates that the plant acquires more resources from aboveground; this may reduce the plant’s carbon cost for AMF and restrict subsequent AMF functions (Johnson, 2010; Konvalinková et al., 2015). Therefore, we assumed that the plant R/S under non-AMF inoculation may play an important role in predicting the effect of AMF on plant growth, and this warrants investigation.

In this study, we conducted a meta-analysis to assess the main biotic and abiotic factors that have an impact on the effect of AMF on plant growth, particularly the RLC and plant R/S. We also collected data regarding the plant family, life history, functional type, root system, and the AMF species number and guild, which were classified by the patterns of the biomass allocation of AMF in roots or soil (Weber et al., 2019). In addition, we also considered the experimental setting, including the soil pH, available phosphorus (AP), soil texture, experimental duration, and pot size. These factors have been routinely considered in other meta-analysis studies on the functions of AMF (Hoeksema et al., 2010; Lehmann et al., 2014; Leifheit et al., 2014; Lehmann and Rillig, 2015; Zhang et al., 2019). In this study, we first examined the significance of these factors on the effect of AMF on plant growth; subsequently, the factors found significant were used to build a model to calculate the importance of the influence of each on the functions of AMF in promoting plant growth.

## Materials and methods

### Data collection

In August 2020, the Web of Science database was used to gather articles using the following the search strings: ‘((shoot AND root) OR (aboveground OR belowground) AND biomass OR R/S OR root/shoot OR (root: shoot) OR (root shoot ratio)) AND (AMF OR (AM fung*) OR arbuscular OR mycorrhiza* OR Glomeromycota)’. The literature search process is detailed in a PRISMA flow diagram in **Supplementary Figure S1**. The mean values of the shoot, root, and total dry biomass, and/or the plant R/S, and the corresponding standard deviation (SD) and sample size (*N*) for AMF-inoculated and non-inoculated plants were extracted from the selected articles. In addition, the R/S data of non-inoculated and/or inoculated (when reported) plants were extracted from the same articles. For a more accurate estimate of the plant R/S, we only investigated studies that were conducted in pot experimental settings. Furthermore, we included only data on the functional organs (shoots and roots) and excluded data on tubers and bulbs. We finally included retrieved articles that fulfilled the following criteria: a) the shoot, root, and/or total dry biomass of AMF-inoculated plants and plants with corresponding control treatments in pot conditions were reported; b) plants from the control treatment had a maximum of 5% AMF RLC and inoculated plants had at least 5% AMF RLC—this criterion could conservatively confirm whether the efficacy of AMF on plant growth was due to the ascertainable colonization (Lehmann and Rillig, 2015); c) each pot experiment was conducted with only one plant species to avoid interspecific competition or facilitation effects between different species; and d) the experimental duration was less than 1 year to avoid root mass accumulation as a result of the aboveground plant parts dying. Combination treatments (AMF inoculation with additional biotic or abiotic treatments) were excluded to avoid possible interactions with AMF. When data were presented only as graphs, we used GetData Graph Digitizer 2.24 (http://getdata-graph-digitizer.com) for data extraction. For articles using two or more plant varieties that did not report the SD or standard error (SE), we calculated the combined mean and SD from the means of the plant varieties. For articles that reported only the SE, SD was calculated as follows: SD = SE * sqrt(*N*). If neither SD nor SE was reported, the SD was calculated as 10% of the mean value (Brown et al., 2014; Zhou et al., 2017). To increase our data coverage, the shoot and root biomass data were combined to determine the total biomass if this was unreported; the SD for the total biomass was calculated according to the Taylor series expansion as follows: ${\text{SD}}_{\text{(total biomass)}}=\text{sqrt}\left({\text{SD}}_{\left(\text{shoot biomass}\right)}^{2}+{\text{SD}}_{\left(root\;biomass\right)}^{2}+2*{\text{COV}}_{\text{(shoot biomass, root biomass)}}\right)$ , where the shoot biomass and root biomass were presumed to be two normally distributed independent variables (Lee and Forthofer, 2006); COV represents the covariance of the shoot and root biomass. For a conservative estimate of the SD of the total biomass, we defined COV as follows: COV = SD_(shoot biomass)_ * SD_(root biomass)_ (by Cauchy–Schwarz inequality).

### Moderator variables

To meet our research target, we chose 13 different moderator variables for their potential to modulate the effects of AMF on the plant shoot, root, and total biomass, as detailed below.

1. Plant root-to-shoot ratio (R/S): We chose the non-inoculated plant R/S as a characteristic that excluded the influence of AMF on plant carbon allocation. The R/S of non-inoculated plants was recorded when reported or was calculated by means of the root and shoot biomass.
2. AMF root length colonization (RLC): This is the infection rate of AMF intraradical hyphae in the plant roots.
3. Host plant family: Families were divided into six categories: Asteraceae, Fabaceae, Poaceae, Rutaceae, Solanaceae, and others. Plant family observations less than 50 were all grouped into “others.”
4. Host plant life history: History was categorized into annual and perennial.
5. Host plant functional type: Functional type was either herbaceous or woody.
6. Root system: Root systems were classified as taproot and fibrous root following the method of Yang et al. (2015).
7. AMF number: Numbers were divided into single and mixed, with mixed-species inoculation comprising more than one AMF species.
8. AMF guild: The guilds for AMF species had four levels—rhizophilic, edaphophilic, ancestral, and mixed—which were classified according to Weber et al. (2019). Briefly, the Glomeraceae, Claroideoglomeraceae, and Paraglomeraceae families belong to the rhizophilic guild, which allocates more biomass to the roots, while Gigasporaceae and Diversisporaceae belong to the edaphophilic guild, which may allocate more biomass to the extraradical hyphae in soil and have a higher ability to promote plant nutrient uptake (Weber et al., 2019); the other remaining families were grouped into the ancestral guild. The mixed level for AMF guild had more than one guild.
9. Pot size: This was a continuous variable that used the soil weight (in kilograms) in the pots. Alternatively, we either converted the provided value to a weight using the bulk density of the soil that was reported or used a bulk soil density of 1.3g/cm^3^, which was reported as a global main bulk soil density measure (Shangguan et al., 2014). We converted the pot sizes into three categories (corresponding weight)—small (<1 kg), medium (1–2.9 kg), and large (>2.9 kg)—with three approximately equal numbers of observations.
10. Soil texture: Texture was either sandy or not sandy. Soils with a sand content of more than 50% was regarded as sandy, while those with less 50% sand content were considered not sandy. These classifications were in accordance with the soil taxonomy of the USDA Natural Resources Conservation Service (http://soils.usda.gov).
11. Soil available phosphorus (AP): Data on the Olsen-extractable P for soil were recorded. When other reagent methods were used for soil AP, these values were converted to the Olsen-extractable P following Wolf and Baker (1985). Subsequently, the soil AP values were classified as either deficient (≤9 mg/kg) or non-deficient (>9 mg/kg) according the definition of Lehmann and Rillig (2015), as P fertilization under ≤9 mg/kg would generally improve plant growth.
12. Soil pH: There were three levels for this moderator—acidic (pH< 6.6), neutral (pH = 6.6-7.3), and alkaline (p> 7.3)—which followed the USDA criteria (http://soils.usda.gov). The main reagent for soil pH was H_2_O; data from the use of other reagents were converted into H_2_O following Lierop (1981). For studies that did not report the reagent, we assumed that H_2_O was used since it is the most commonly used reagent. We used the pH–H_2_O data for this variable.
13. Experimental duration: This was used as a continuous variable. We only considered the duration from when plants were inoculated with AMF. Hoeksema et al. (2010) showed that the plant tissue N/P also influences the functions of AMF, but this was not reported in the majority of the studies in our dataset. Thus, we did not collect the plant N/P data for this study. To meet normality, data on the RLC, R/S, and the experimental duration were square-root (sqrt) transformed.

### Data analysis

All analyses were performed using R-4.1.3 (R Core Team, 2016). To measure the effect size of AMF, we calculated the response of plants to AMF inoculation as the log response ratio (RR) (Hedges et al., 1999), as follows: RR = $\ln\left(\frac{X_{\text{t}}}{X_{\text{s}}}\right)$ , where *X*_t_ is the mean plant biomass (the shoot, root, and total biomass) of plants inoculated with AMF and *X*_s_ is the mean plant biomass in the control treatment. The variance (Var) of each RR was calculated as: $\text{Var}\left(\text{RR}\right)=\frac{S_{\text{t}}^{2}}{n_{\text{t}}X_{\text{t}}^{2}}+\frac{S_{\text{c}}^{2}}{n_{\text{c}}X_{\text{s}}^{2}}$ , where *n*_t_ and *n*_c_ are the sample sizes of the AMF inoculation and the control groups, respectively, while $S_{\text{t}}^{2}$ and $S_{\text{c}}^{2}$ are the SDs of the AMF inoculation and the control treatment, respectively (Gurevitch et al., 2001). In this study, a random effects model was used to calculate the weighted response ratio (RR_++_) and the 95% confidence intervals (CIs) using the *rma* function in the “metafor” package (Viechtbauer, 2010). If the 95% CIs did not overlap with zero, the RR_++_ was considered significant (*p*< 0.05). Observations that had any RRs on the shoot, root, or total biomass with a standardized residual value over the absolute value of 3 were removed as extreme values (Anton et al., 2019; Batastini et al., 2021). A failsafe-*N* analysis was used to assess the potential for publication bias that will impact our results (Rosenberg, 2005). The failsafe numbers for the shoot, root, and total biomass were 37,208,645, 37,966,268, and 86,730,903, respectively (**Supplementary Figure S2**). These were all >39-fold the threshold of 8,210, indicating no potential publication bias in our results.

We used the *rma* function to fit a random effects model in order to test the influence of moderators on the RR_++_, the significance of the correlations between RR and the continuous moderators, or the differences between any two levels of each moderator. For this step, the response variable was RR, the fixed effects were the moderators, and the variance was the Var(RR). This method was also used to examine the differences between the RRs on the shoot, root, and total biomass. To visualize the results of the differences between the levels of the moderators, we calculated the RR_++_ and 95% CI for each level separately. To determine the importance of the moderator that significantly influenced the variations in the effect size of AMF on plant growth, a weighted random forest analysis was conducted using the *MetaForest* function in the “MetaForest” package (Van Lissa, 2017; Byrnes et al., 2018).

## Results

**Figure 1:**
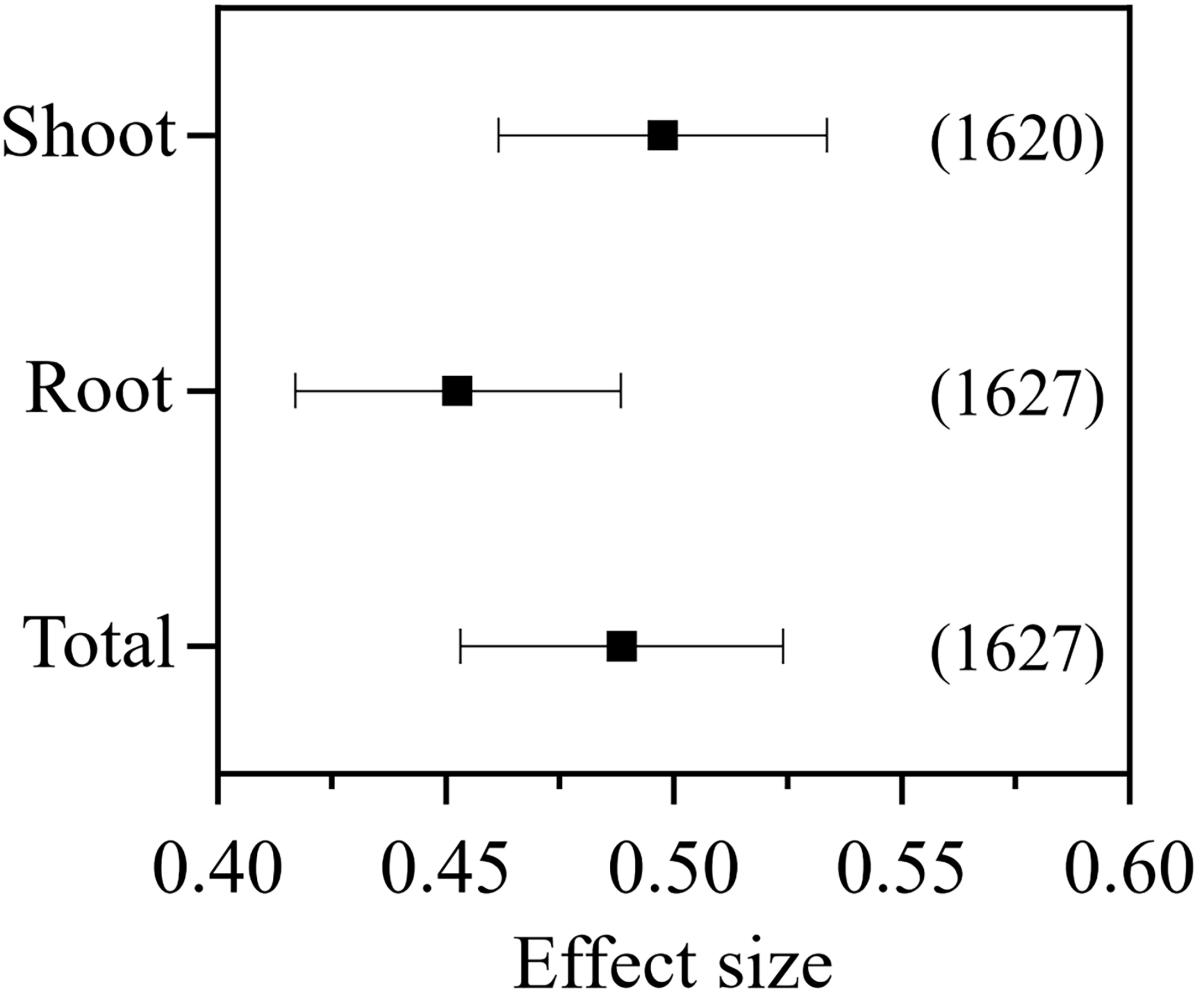
Effect of arbuscular mycorrhizal fungi (AMF) inoculation on the plant shoot, root, and total biomass.

In total, 639 published studies met the criteria, which included 1,640 independent observations (**Supplementary Data File S1**). After removal of the outliers, 1,620, 1,627, and 1,627 observations were retained for the meta-analysis of the shoot, root, and total biomass, respectively. The inoculation of AMF in pot experiments positively affected the plant shoot (RR_++_= 0.498, 95% CI = 0.461–0.533), root (RR_++_ = 0.453, 95% CI = 0.417–0.488), and the total biomass (RR_++_ = 0.488, 95% CI = 0.453–0.524) (**Figure 1**).

**Figure 2:**
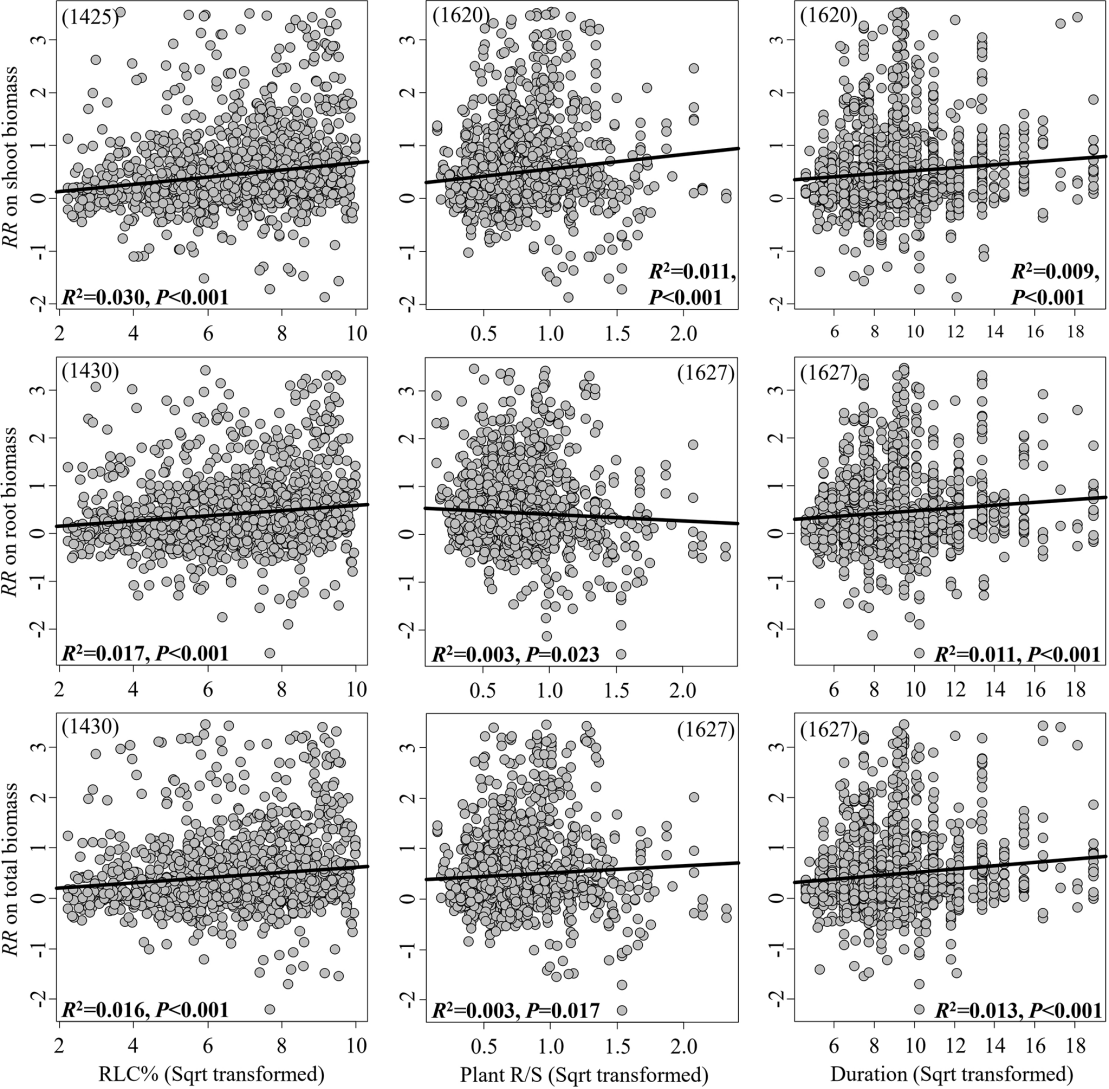
Linear relationships between the effect size of arbuscular mycorrhizal fungi (AMF) on plant biomass (shoot, root, and total) and the AMF root length colonization (RLC), plant root-to-shoot ratio (R/S), and experimental duration. The significance test for the linear relationship was based on a random effects model with a residual maximum likelihood (REML) method. *P*-values ≤0.05 were considered significant. *Values in parentheses* are the number of observations included in the analysis.

**Figure 3:**
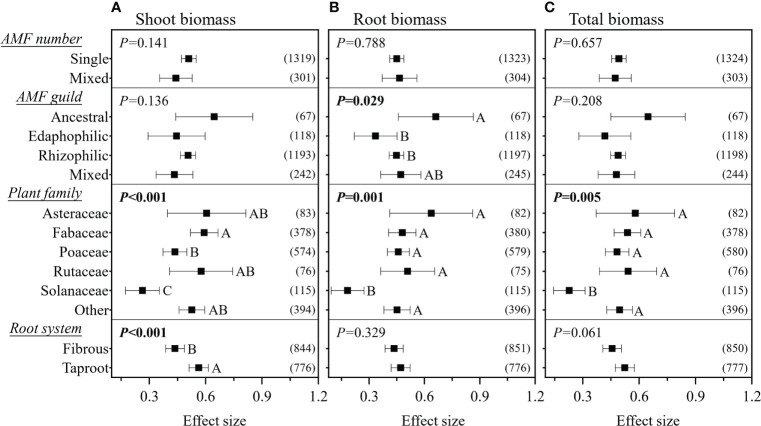
Effect of arbuscular mycorrhizal fungi (AMF) number and guild, plant family, and root system on the effect size of AMF on plant shoot **(A)**, root **(B)**, and total **(C)** biomass. *Values in parentheses* are the number of observations included in the analysis. The significance test for between-level differences was based on a random effects model with a residual maximum likelihood (REML) method. *P*-values ≤0.05 were considered significant. *Different capital letters* indicate significant differences compared with the other groups as assessed using a random effects model with a REML method.

**Figure 4:**
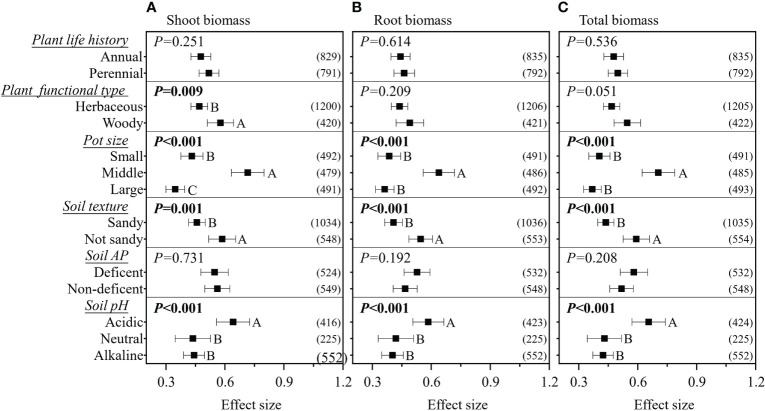
Effect of plant life history, plant functional type, pot size, soil texture, soil available phosphorus (AP), and soil pH on the effect size of arbuscular mycorrhizal fungi (AMF) on plant shoot **(A)**, root **(B)**, and total **(C)** biomass. *Values in parentheses* are the number of observations included in the analysis. The significance test for between-level differences was based on a random effects model with a residual maximum likelihood (REML) method. *P*-values ≤0.05 were considered significant. *Different capital letters* indicate significant differences compared with the other groups as assessed using a random effects model with a REML method.

The RRs on the shoot, root, and total biomass significantly increased in response to the continuous moderators RLC and experimental duration, while the RRs on the shoot and total biomass significantly increased in response to plant R/S; in contrast, the RR on root biomass significantly decreased (**Figure 2**). However, all these linear relationships were weak, with *R* ^2^< 0.1. In pot experiments, inoculation with mixed AMF species had no higher effect on plant biomass (**Figure 3**). The AMF guild had significant influence only on the RR_++_ on root biomass, of which ancestral AMF species showed a greater increase in root biomass compared to edaphophilic and rhizophilic AMF species (**Figure 3B**). Plant family showed a significant influence on the RR_++_ on shoot, root, and total biomass. For all three RR_++_, that of AMF in Solanaceae plants was lower. In addition, the RR_++_ on shoot biomass in Poaceae plants was significantly lower than that in Fabaceae (**Figure 3A**). Both root system and plant functional type affected only the RR_++_ on shoot biomass, of which taproot or woody plants had higher values (**Figures 3**, **4**). Annual and perennial plants showed no significantly different responses to AMF (**Figure 4**). The pot experimental setting, including the pot size, soil texture, and soil pH, but not soil AP, had significant effects on all three RR_++_. The analysis found that AMF had higher RR_++_ on the shoot, root, and total biomass in medium-sized pots, not sandy soils, or acidic conditions (**Figure 4**).

**Figure 5:**
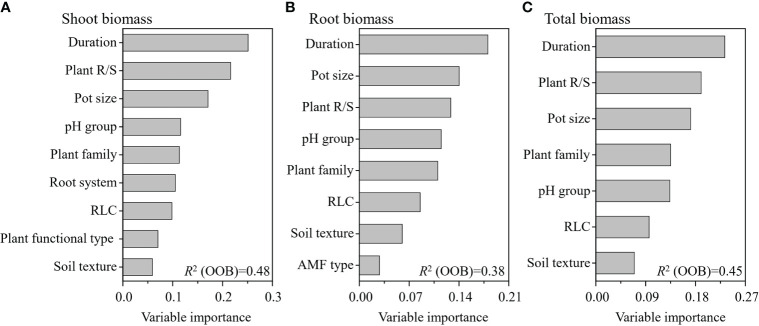
Variable importance of the moderators for the effect of arbuscular mycorrhizal fungi (AMF) on plant shoot **(A)**, root **(B)**, and total **(C)** biomass. This weighted random forest analysis included the significant moderators in the previous random effects model.

The weighted random forest analysis showed that experimental duration was the most important moderator of the RR on the shoot, root, and total biomass (**Figure 5**). The second most important moderator was plant R/S for both shoot and total biomass, while it was pot size for root biomass. The third most important moderator was pot size for shoot and total biomass, while it was plant R/S for root biomass (**Figure 5**). The weighted random forest analysis clearly revealed that the experimental duration, plant R/S, and pot size were the three most important moderators determining the effects of AMF on the shoot, root, and total biomass.

## Discussion

Previous studies found that the effect size of AMF on soil aggregation (Leifheit et al., 2014) and iron concentration (Lehmann and Rillig, 2015) was higher at the intermediate duration. However, we found that the effect of AMF on plant growth increased with experimental duration in the individual moderator test (**Figure 2**); moreover, the random effects model confirmed that the experimental duration was the most important moderator when also considering other moderators. The main reason for this is the fact that soil aggregation or plant iron would be depleted over time, while the biomass keeps accumulating, even if the benefit of AMF decreases due to the establishment of the plant root system or pot-bound roots at a later duration, which also leads to the effect size on biomass slowly increasing with experimental duration. This increasing positive effect with experimental duration could also occur in any pot experimental setting, which might explain why experimental duration was the most important moderator.

Plants with a higher R/S showed greater increases of both shoot and total biomass as a result of AMF inoculation, and this is consistent with our assumptions. Surprisingly, R/S was negatively correlated with the effect of AMF on the root biomass (**Figure 2**). It is possible that, due to the more biomass plants allocated to roots (a high R/S) for soil resources, AMF played a stronger functional compensatory role, thereby alleviating the biomass allocation to the roots. Furthermore, our results on total biomass contradicted those of Azcón and Ocampo (1981) and Johnson (2010), which showed a negative correlation between plant R/S and the effect of AMF on total biomass. We believe in the higher reliability of our results since we used a very large database that included numerous factors (more than 100-fold that of Azcón and Ocampo). Earlier studies have revealed that environmental (Hoeksema et al., 2010) and evolutionary (Anacker et al., 2014) constraints can impact the influence of AMF on plant growth and can also directly determine a plant’s biomass allocation strategy (Hodge, 2004; Poorter et al., 2012b). Therefore, besides experimental duration, a definite plant R/S may be the most basic indicator of the influence of abiotic and biotic factors on the role of AMF, particularly for shoot and total biomass.

Despite both experimental duration and plant R/S being important in the prediction of the effect size of AMF on the plant shoot, root, and total biomass, we found that the correlations of these two moderators with the effect size were all weak. This might be due to the numerous factors that can influence the functions of AMF, including plant and/or AMF species (Marro et al., 2022), plant nutrition status, and experimental light intensity (Johnson, 2010). Accordingly, these weak relationships might be the result of the different experimental factors, which should be taken into consideration in future research.

We found that experiments using medium-sized (1–2.9 kg) pots showed a higher effect size than when using small and large pot sizes (**Figure 4**). In small pots, the roots could more easily be pot-bound at the same experiment duration; on the other hand, plants in small pots also have lower photosynthesis rates, according to the results of Poorter et al. (2012a); these two conditions may have reduced the function of AMF in small pots (Johnson, 2010). For plants in large pots, the root mass was relatively smaller than that in those planted in small and medium pots, which could be due to an increase in the amount of nutrients and water available for the plant, reducing the plant’s dependence on AMF (Poorter et al., 2012a). The weighted random forest analysis further revealed that pot size played very important roles in determining the effect size when considering other moderators, especially for root biomass. These results highlight the need for careful consideration of the pot size in inoculation experiments with AMF.

The relationship between RLC and the effect of AMF on plant biomass has been investigated previously (Treseder, 2013; Zheng et al., 2013). Our results confirmed that the effects on plant shoot, root, and total biomass increased with RLC. RLC directly determines the nutrient exchange ratio between plants and AMF (Smith et al., 2009; Bücking et al., 2012; Simó-González et al., 2019); however, our results did not support RLC being the most important moderator predicting the effects of AMF on plant biomass based on such a large dataset across different experimental settings and biotic and abiotic factors. Generally, the relationship between RLC and experimental duration is not linear but curved (Antonio and Nogueira, 2003; Samiappan and Manian, 2009), which demonstrates that RLC may have better predictive value on effect size at a relatively short time duration, but not the entire duration, especially when compared to the effect of experimental duration.

In this study, we did not find any significant difference between the effects of single and mixed AMF inoculations on plant biomass (**Figure 3**). The mixed inoculum was expected to show a higher increase in biomass by increasing the probability of the presence of beneficial species or the inclusion of species that have complementary functions (van der Heijden et al., 1998; Hart and Reader, 2002; Wagg et al., 2011). It can be explained that not all AMF species are functionally complementary, but may be functionally redundant, and even some mixed inoculations could increase the carbon competition among AMF species (Liu et al., 2015). Weber et al. (2019) made the assumption that edaphophilic AMF would have a greater effect on the promotion of plant nutrient uptake and biomass, but our findings did not support this. We discovered that AMF guilds only had a significant influence on the effect of AMF on root biomass (**Figure 3B**) and that ancestral AMF had a higher effect size than rhizophilic and edaphophilic AMF guilds. The reason for this is unclear, but we theorized that it may be due to the ancestral AMF having a balanced biomass distribution between the roots and soil, resulting in an optimal function in promoting plant growth (Weber et al., 2019). Another possibility could be insufficient sampling, particularly for the ancestral and edaphophilic guilds; hence, more research is required in this field.

Our results demonstrated that AMF had a lower effect size on the shoot, root, and total biomass of plants in the Solanaceae family compared to those in other families (**Figure 3**), similar to the results on crops from the study of Van Geel et al. (2016). The reason for this is unclear, and this should be investigated with regard to the physiological and biochemical interactions between AMF and Solanaceae plants. Furthermore, we confirmed that the shoot biomass of Poaceae plants was less affected by AMF compared to Fabaceae plants. As these two plant families are generally used as host plants in AMF studies, careful comparison should be done in further studies. We found that AMF had a lower effect size on the shoot biomass of fibrous root plants, which is supported by the results of Yang et al. (2015), as fibrous root plants have more fine roots and root hairs that directly absorb soil nutrients rather than through AMF. We also found that plant functional type had a significant impact on the effect size on shoot biomass than plant life history. This could be explained by two aspects: 1) woody plants could show a larger effect of AMF because their taproot systems are more dependent on AMF, as found in this study and in previous studies (Hetrick et al., 1988; Tawaraya and Keitaro, 2003; Yang et al., 2015); 2) the life history strategy of perennial herbaceous plants (excluding woody perennial plants from perennial plants) ensures sufficient root biomass, rather than shoot biomass, for future plant shoot regrowth, making the shoot biomass of perennial herbaceous plants being less impacted by AMF (Boerner, 1992).

AMF had lower effects on plant biomass in sandy soils. This is due to sandy soils having a lower adsorption capacity and, therefore, an enhanced mobility and solubility of the soil micronutrients, flushing the available nutrients and water more quickly (Najafi-Ghiri et al., 2013). On the other hand, sandy soils are beneficial to root growth due to the lower pressure on the roots (Dexter, 2004), which may increase the absorption of nutrients by plant roots and may reduce AMF hyphae. The soil AP had no impact on the effect of AMF on plant biomass, even with the soil P being confirmed as the primary factor maintaining the symbiosis between AMF and plants (Smith and Read, 2008; Kiers et al., 2011). This is mainly due to the soil stoichiometry for N/P, rather than only AP, having a more significant influence on the functions of AMF (Johnson, 2010; Han et al., 2020). Unfortunately, these data were not reported in most studies and require further investigation. We found that the soil pH had an impact on the effect size on the shoot, root, and total biomass, showing that AMF had a higher effect size in acidic soils (**Figure 4**). This is caused by the normally major nutrient deficiencies in acidic soils (Neina, 2019) that could increase the plants’ dependence on AMF (Johnson, 2010).

## Conclusion

This meta-analysis based on 639 studies with 1,640 observations confirmed that AMF inoculation increased the plant shoot, root, and total biomass. At present, this is the first study that quantitatively synthesized the importance of factors influencing the effects of AMF inoculation on plant biomass. Our results provided insights into the effects of AMF on plant biomass mainly depending on the experimental duration, plant carbon allocation strategy (R/S), and experimental pot size when considering the effects of other experimental settings and other abiotic and biotic factors in pot experiments. Clarifying these main predictive factors could help in understanding the role of AMF in promoting plant growth and in revealing their real functions by avoiding the superposition of unfavorable factors. In this study, we also found that both experimental duration and plant R/S had weak relationships with the effect size, which indicates that these relationships might be influenced by numerous other factors, which should be considered in future research.

As a study limitation, we only investigated the effect of AMF inoculation on plant growth at a single plant species level. This condition is equivalent to an agro-ecosystem with a single crop species. The main AMF inoculum included only a single species; however, in natural ecosystems, on average, host plants can harbor up to 30 AMF species (Kivlin et al., 2011). Therefore, multiple AMF species and their inocula should be taken into account to simulate natural ecosystems. Our study primarily considered the effect of individual AMF species, but in natural ecosystems, various interactions and/or cooperation from other groups of microbes, such as N-fixing microbes (Miransari, 2011), soil pathogens (Harrier and Watson, 2004; Qin et al., 2021), and even the native AMF community (Rodriguez and Sanders, 2014), exist. Accordingly, more research should be conducted in the future to better predict the factors that impact the functions of AMF under natural environmental conditions.

## Data availability statement

The original contributions presented in the study are included in the article/**Supplementary Material**. Further inquiries can be directed to the corresponding author.

## Author contributions

MQ and LL designed the study with the help of WW and YT. MQ, LL, WW, and BS analyzed the data. MQ, J-PM, and MO wrote the manuscript, with extensive discussions with YT and WW. All authors contributed to revisions. All authors contributed to the article and approved the submitted version.

## Funding

We are grateful to the financial support of the Second Tibetan Plateau Scientific Expedition and Research Program (STEP; grant no. 2019QZKK0301); the Fundamental Research Funds of China West Normal University (19E048 and 18Q046); the National Natural Science Foundation of China (31870579, 31870494, and 31971445); and the Applied Basic Research Program of Sichuan Province (2020YJ0346).

## Conflict of interest

The authors declare that the research was conducted in the absence of any commercial or financial relationships that could be construed as a potential conflict of interest.

## Publisher’s note

All claims expressed in this article are solely those of the authors and do not necessarily represent those of their affiliated organizations, or those of the publisher, the editors and the reviewers. Any product that may be evaluated in this article, or claim that may be made by its manufacturer, is not guaranteed or endorsed by the publisher.
