# Supplementary material for: Experimental duration determines the effect of arbuscular mycorrhizal fungi on plant biomass in pot experiments: A meta-analysis
Source: Front Plant Sci. 2022 Nov 3;13:1024874. doi: 10.3389/fpls.2022.1024874 (PMC9671359; doi:10.3389/fpls.2022.1024874)

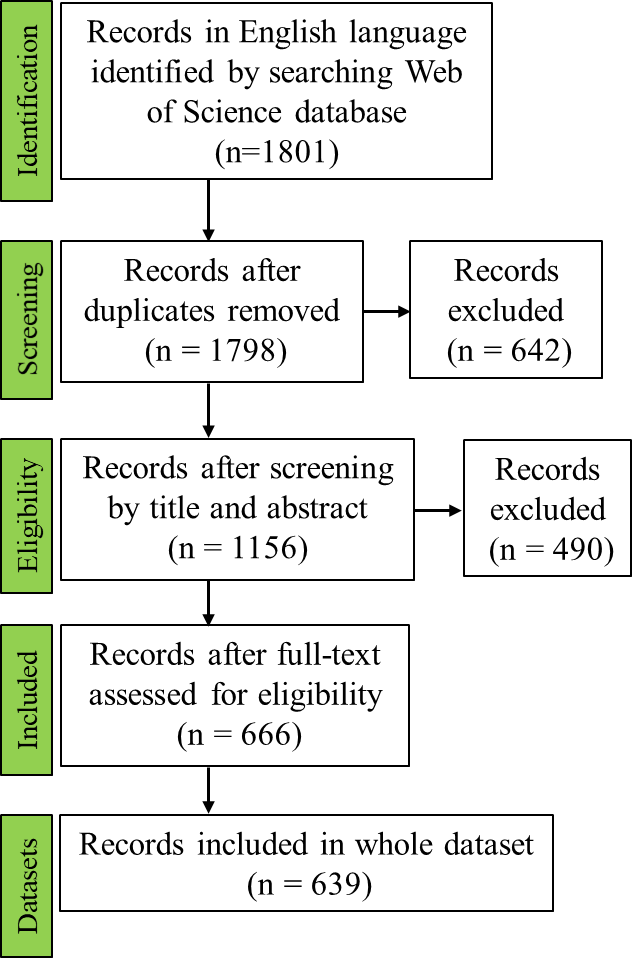


Fig. S1 PRISMA diagram showing the process of locating publications for this meta-analysis.

Fig. S2 Funnel plots for observed outcomes of plant shoot, root, and total biomass under AMF inoculation in pot experiments.


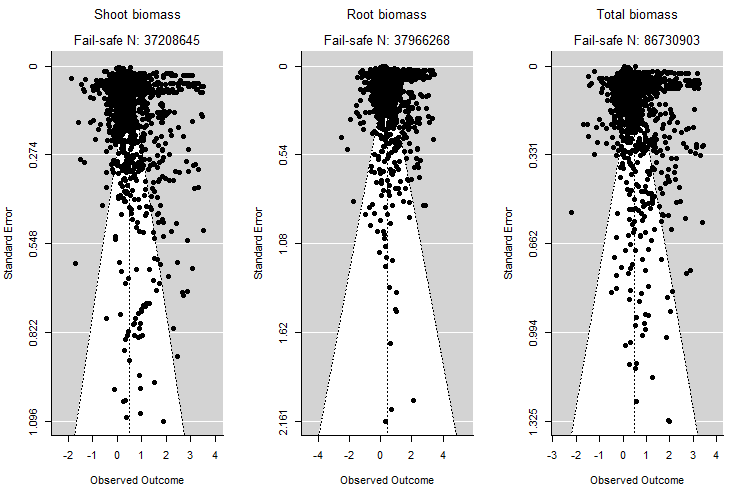

Supplement: Supplementary file 2 [file DataSheet_2.docx]
